# Extrusion-Cooking Modifies Physicochemical and Nutrition-Related Properties of Wheat Bran

**DOI:** 10.3390/foods9060738

**Published:** 2020-06-04

**Authors:** Chiara Roye, Muriel Henrion, Hélène Chanvrier, Karlien De Roeck, Yamina De Bondt, Inge Liberloo, Roberto King, Christophe M. Courtin

**Affiliations:** 1 Laboratory of Food Chemistry and Biochemistry and Leuven Food Science and Nutrition, Research Centre (LFoRCe), KU Leuven, Kasteelpark Arenberg 20, 3001 Leuven, Belgium; karlien.deroeck@kuleuven.be (K.D.R.); yamina.debondt@kuleuven.be (Y.D.B.); inge.liberloo@kuleuven.be (I.L.); christophe.courtin@kuleuven.be (C.M.C.); 2 Société des Produits Nestlé S.A., Nestlé Research and Development Orbe, Route de Chavornay 3, 1350 Orbe, Switzerland; Muriel.henrion@rdor.nestle.com (M.H.); Helene.chanvrier@rdor.nestle.com (H.C.); 3 Société des Produits Nestlé S.A., Nestlé Research, Vers-chez-les-Blanc, 1026 Lausanne, Switzerland; rokisol@hotmail.com

**Keywords:** extrusion-cooking, wheat bran, in vitro fermentation, phytate, ferulic acid, screw configuration, bran–water interaction

## Abstract

The potential of extrusion-cooking to change the physicochemical characteristics of wheat bran, increase its nutritional value and decrease its recalcitrance towards fermentation was investigated in this study. The conditions in a twin-screw extruder were varied by changing screw configuration, moisture content and barrel temperature. The former was not previously investigated in studies on bran extrusion. Extrusion-cooking resulted in an increased water-holding capacity and extract viscosity of bran, suggesting shear-induced structure degradation and structure loosening due to steam explosion at the extruder outlet. Modelling showed that the extent of these modifications mainly correlates with the amount of specific mechanical energy (SME) input, which increases with an increasing number of work sections in the screw configuration and a decreasing moisture content and barrel temperature. Extrusion led to solubilisation of arabinoxylan and ferulic acid. Moreover, it led to starch melting and phytate degradation. Upon fermentation of the most modified sample using a human faecal inoculum, small numeric pH decreases and short-chain fatty acid production increases were observed compared to the control bran, while protein fermentation was decreased. Overall, extrusion-cooking can improve the nutrition-related properties of wheat bran, making it an interesting technique for the modification of bran before further use or consumption as an extruded end product.

## 1. Introduction

Health awareness in society is increasing, and significant knowledge about the importance of dietary fibre in a healthy diet is available [1,2]. Unfortunately, in most countries, the actual daily dietary fibre intake [1] is lower than the recommended intake [3]. Consequently, dietary fibre consumption should be increased. One way to achieve this is by adding more dietary fibre to food products.

Wheat bran is commercially available as a by-product of the grain milling process and consists of the botanical bran and the aleurone with some residual endosperm attached to it. Two health claims are approved by the European Food Safety Authority with regard to the consumption of wheat bran fibre. It can contribute to an increase in faecal bulk and can decrease—or, more correctly, regulate—the intestinal transit time [3]. These effects can be mostly attributed to the physical presence of the dietary fibre fraction of bran in the large intestine as it is not digested in the small intestine [4]. The water-binding capacity of bran, caused by the capillary structure of the tissue and the large polymer networks constituting the bran cell walls, also contributes to the effect [5]. In addition, fermentation of the soluble dietary fibre fraction, and to a more limited extent, the insoluble dietary fibre fraction, lead to the production of short-chain fatty acids (SCFA), to which different positive health effects are attributed [6,7,8].

Despite the health benefits associated with wheat bran consumption, it can be questioned whether native millers’ bran can exert its optimal physiological and nutritional impact when ingested in light of the structure and properties of wheat bran. Firstly, the bran structure is rigid. Cellulose and arabinoxylan in wheat bran are known to be recalcitrant due to their strong associations with other bran compounds and their high molecular weight [9,10]. In addition, the bran structure itself is possibly not very accessible for gut microbiota. It is suggested that an accessible bran structure can act as a sort of “dinner table” to which bacteria can adhere to ferment soluble and insoluble dietary fibre efficiently [11,12]. Secondly, bioactive compounds are entrapped inside rigid aleurone cell walls which withstand digestion in the gastro-intestinal tract [11], limiting their absorption. Thirdly, the presence of phytate in wheat bran can decrease the mineral bioaccessibility, as phytate chelates divalent minerals such as Zn^2+^ and Fe^2+^ [12]. Finally, ferulic acid is the most abundant potentially beneficial phytochemical in wheat bran, with 90% bound to arabinoxylan and lignin [13,14]. Only the unbound fraction is absorbed in the small intestine and can exert its beneficial effects as an antioxidant in our body [15]. Modification strategies that can decrease the recalcitrance and compactness of the wheat bran structure and improve the accessibility of minerals and bioactives are highly desirable. Possible strategies include particle size reduction, bioprocessing and extrusion-cooking.

Extrusion-cooking is a commonly used process for the production of breakfast cereals and expanded snacks that subjects ingredients to high pressures, high temperatures and high shear forces for a short time. An extruder consists of one (single screw) or two (twin screw) rotating screws in a barrel with a small opening (die) at the exit [16]. The dry ingredients, together with the desired amount of water, are added and mixed due to the rotating action of the screw. The intensity of the mixing, and thus the amount of shear, depends on the geometry of the screw [17]. During extrusion-cooking, many structural changes and physicochemical transformations of food constituents take place, such as melting and depolymerisation of starch, protein denaturation and lipid oxidation. The extent of these transformations depends on the intensity of the extrusion-cooking process [18,19], which can be varied by changing process parameters. In addition, raw material characteristics will also determine the transformations.

Studies about wheat bran extrusion-cooking are only scarcely present and mainly focus on the hydration properties of extruded wheat bran and the solubility of dietary fibre. Ralet et al. [20] observed an increase in the water-absorption speed, but no effect on the total water-binding capacity. On the contrary, Yan et al. [21] observed an increase in the water-binding capacity of wheat bran after extrusion-cooking. Moreover, it was shown that dietary fibre was solubilised during extrusion-cooking [20,21,22] due to the conversion of water-unextractable arabinoxylan (WU-AX) to water-extractable arabinoxylan (WE-AX) and the release of β-glucan [22,23].

The aim of this article was to gain insight into the effect of screw configuration, moisture content and barrel temperature during extrusion-cooking on wheat bran characteristics. A systematic study of the effect of changing screw configuration on the processing of wheat bran and wheat bran characteristics has not yet been reported in the literature, and is novel in this article. The extent to which the physicochemical properties of wheat bran can be modified with extrusion-cooking was also investigated, and this in-depth characterisation of extruded wheat bran is innovative compared to the existing literature. Attention was paid to the effect of extrusion-cooking on bran–water interactions, degradation of the wheat bran structure, increase in extractable dietary fibre content, and finally degradation of phytate and release of ferulic acid.

## 2. Materials and Methods

### 2.1. Materials

Native wheat (*Triticum aestivum* L.) bran was provided by Dossche Mills (Deinze, Belgium) and characterised. Methods for characterisation of the bran and composition are discussed in Roye et al. [24]. The miller’s wheat bran contained 11.8% starch, 20.9% proteins, 5.4% lipids, 24.4% WU-AX, 0.6% WE-AX, 9.1% cellulose, 2.1% β-glucan, 3.3% fructan, 4.3% phytate and 6.4% ash on dry matter (dm) basis [24]. The average particle size of the bran material was 1421 µm. Chemicals, solvents and reagents were purchased from Sigma-Aldrich (Bornem, Belgium).

### 2.2. Extrusion-Cooking

**Figure 1:**
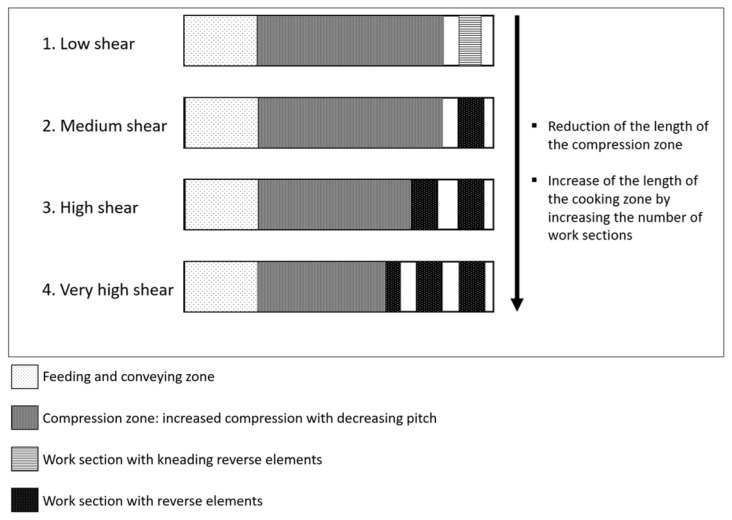
Graphical representation of the low shear, medium shear, high shear and very high shear screw configurations in the extruder.

The native wheat bran was extruded in a BC21 twin-screw extruder (Clextral, France). Four different screw configurations were designed (Figure 1). Every screw configuration consisted of a feeding and conveying zone at the beginning, followed by a compression zone. In this zone, compression was increased due to a decreasing pitch, defined as the distance between two consecutive flights. The length of the cooking zone and the number of work sections with reverse elements differed for every configuration, leading to four different screw configurations. They were denoted as a low shear (LS) screw configuration, a medium shear (MS) screw configuration, a high shear (HS) screw configuration and a very high shear (VHS) screw configuration. It has to be remarked that the LS screw configuration already consisted of a kneading reverse element and thus already applied a considerable amount of shear. The extruder itself consisted of 5 consecutive barrels and measured 500 mm in length. Every barrel was equipped with individual temperature control. The screw diameter was 25 mm (centerline distance: 21 mm), the orifice of the die was circular and measured 3 mm. The feed rate of wheat bran was set at 7 kg/h. During the extrusion-cooking experiments, maximally achievable levels of shear and temperature were targeted stepwise.

An overview of the samples produced is shown in Table 1. Extrusion-cooking experiments were conducted in singlicate as the study is an exploratory study and priority was allocated to producing samples using different process parameters. Formulations with a total moisture content of 23%, 27% and 33% were used. Water was injected directly into the extruder. Twenty-three percent of moisture was the lowest moisture content that could be used while keeping the production process stable. Lowering the moisture content further led to blockage of the extruder or steam formation within the extruder, resulting in pressure and temperature fluctuations. Two different temperature profiles were used: a low-temperature profile (20 °C, 80 °C, 105 °C, 115 °C and 120 °C in the consecutive barrels, from feeding zone to extruder exit order) and a high-temperature profile (20 °C, 90 °C, 120 °C, 135 °C and 145 °C). The high-temperature profile uses the highest temperature that could be reached in the last barrel while keeping the production process stable. Not all combinations of screw configuration, moisture content and barrel temperature were produced. The screw speed was kept constant at 310 rpm for all runs. During extrusion-cooking, the die pressure, the specific mechanical energy (SME) and the product temperature were measured. The SME is the mechanical energy input (work) from the drive motor into the material [25]. SME was determined by measuring the torque (Nm) at a constant screw speed (310 rpm) using the following formula: SME = [2 × π × Torque × Screw speed]/flowrate of the product. The pressure was measured in the last barrel before the product exits the extruder and will be referred to as outlet pressure. The product temperature, i.e., the temperature in the last barrel, reflects the highest temperature that the product experienced during the extrusion-cooking process. Overall, the product temperature in barrel one, two, three and four reached about the same value as the set temperature (results not shown). Large differences between the set and the actual temperature are seen in the fifth barrel because of heat dissipation by shear, as high-shear elements were added to this section of the screw. The residence time of the material in the extruder was measured using beetroot powder. It was estimated to be between 34 and 95 s when the screw speed was set at 310 rpm, and the very high shear screw configuration was used. The material exited the extruder as a strand with a diameter of approximately 8 mm.

After extrusion-cooking, samples were dried in a Minimat convection oven (Wiesheu, Großbottwar, Germany) for 20 min at 120 °C, such that the moisture content was brought below 3%. Part of the extruded product was used as such for visualisation of the macroscopic structure of the strand. Prior to physicochemical characterisation, extruded wheat bran was milled to particles with a D50 (median volume-weight value) of 350 to 400 µm with a Cyclotec 1093 Sample Mill (FOSS, Högenäs, Sweden) using a 0.8 mm sieve. Native bran was included in the experiments as a control sample. It was dried in the convection oven for 20 min at 120 °C to exclude the effect of drying on the results obtained and was milled with the Cyclotec 1093 Sample Mill with a sieve of 0.5 mm to obtain a sample with a D50 comparable to the extruded samples.

### 2.3. Structure Visualisation

Light microscopy was used to visualise the structure of selected extruded wheat bran samples (LS23, VHS23), as described in Roye et al. [24]. Starch was coloured purple with a Lugol solution, and proteins were coloured green with a light green solution. X-ray tomography scans of the unmilled extruded products were made with a Skyscan 1172 MCT (Kontich, Belgium) with an X-ray beam of 40 kV and 100 μA as described in Chanvrier et al. [26].

### 2.4. Determination of the Strong Water-Binding Capacity and Extract Viscosity

The strong water-binding capacity (SWBC) of the samples, defined as the amount of water that remains bound to soaked bran when an external force is applied and when drained water and bran are not in contact afterwards [27], was determined as described in Roye et al. [24]. For extract viscosity determination, 4.0 g of native or extruded wheat bran was added to 30.0 mL deionised water. The solution was shaken (150 rpm, 30 min, room temperature) and centrifuged (4000× *g*, 10 min, room temperature). Extract viscosity was measured on the supernatant using an HR-2 Discovery Hybrid Rheometer with a Standard Peltier Concentric cylinder and a DIN Rotor (TA Instruments, New Castle, DE, USA). Viscosity was measured as a function of shear rate between 10/s and 100/s at 20 °C (in duplicate). Since viscosity did not vary with shear rate (Newtonian behaviour), viscosity was expressed as the average viscosity of the replicates at the different shear rates.

### 2.5. Chemical Composition of Extruded Wheat Bran

The method for the determination of the phytate content was described in Roye et al. [24]. The amount of enzymatically degradable starch was determined using the starch damage assay kit of Megazyme (Bray, Ireland), according to AACC International method 76-30.02 [28]. Peroxidase activity was measured as described in Jacobs et al. [27]. All analyses were conducted in triplicate.

Free ferulic acid content was determined using the procedure of Li, Shewry and Ward [29] with some adaptations. Aqueous MeOH (1.0 mL; 70%) was added to 50.0 mg of sample. The solution was vortexed, sonicated (5 min) and centrifuged (5 min, 5000 rpm). The supernatant was recovered, and the extraction procedure was repeated twice on the pellet. The extracts were combined and 100 µL internal standard was added (35 mg 3,5-dichloro-4-hydroxybenzoic acid in 25 mL of aqueous MeOH (40% *v*/*v*) and diluted 20 times with water) before overnight drying with a Speedvac (Fisher Scientific AG, Wiltrasse, Switzerland). An amount of 500 µL of MeOH (40% *v*/*v*) was added to the dried residue, before centrifugation and filtration (cut-off of 3 kDa). The supernatant was transferred to a vial and analysed with an Ultimate 3000 HPLC (Thermo Fisher Scientific, Waltham, MA, USA) with diode array detection set at 320 nm. The separation was performed using an XBridge C18 column (3.0 × 150 mm) (Waters, Wexford, Ireland). The temperature of the column was set at 30 °C, and a gradient elution program with 1% (*v*/*v*) orthophosphoric acid and acetonitrile was used at a flow rate of 0.5 mL/min. Sample (20 µL) was injected, and ferulic acid content was determined using the response of the internal standard and using a calibration curve for this standard. The calibration curve was made using solutions of trans-ferulic acid (C10H10O4 1287.08-25G) and internal standard (3,5-Dichloro-4-hydroxybenzoic D64007-25G) in MeOH (40% *v*/*v*). The free ferulic acid content analysis was performed in duplicate.

### 2.6. Chemical Composition of Wheat Bran Extract

Water extractable (WE) compounds were determined on an aqueous bran extract, prepared with 1.00 g of wheat bran and 30.0 mL of water. The mixture was shaken (150 rpm, 30 min, room temperature), centrifuged (4000× *g*, 10 min, room temperature) and decanted. Extractability was determined, as described in Roye et al. [24].

The amount of WE-AX and WE polymeric glucose was determined with gas chromatography using the method of Englyst and Cummings [30], with some adaptations as described in Gebruers et al. [31]. The AX content in the extract was determined as the sum of arabinose and xylose multiplied by 0.88, to account for the polymeric nature of AX. The arabinose to xylose ratio (A/X) was calculated as a measure of the substitution degree of AX. The amount of WE polymeric glucose was calculated as the amount of glucose multiplied by 0.9 to correct for the polymeric nature of the molecules. The average degree of polymerisation (DP) of WE-AX and WE polymeric glucose was calculated after determination of the amount of reducing end sugars in the extract, using the procedure developed by Courtin and coworkers [32]. The average DP of WE-AX was determined as the sum of arabinose and xylose, divided by the amount of reducing end xylose. The average DP of polymeric glucose was determined as the amount of polymeric glucose in the extract divided by the amount of reducing end glucose in the extract. The amount of free sugars was determined based on the method of Englyst and Cummings [30] as modified by Courtin et al. [32]. The method to determine WE-β-glucan content was based on the Megazyme Mixed Linkage β-Glucan assay (Bray, Ireland), with some adaptations to account for the liquid nature of the sample. An amount of 1.0 mL of extract was transferred to a glass tube, and 0.2 mL of ethanol (50%) and 2.8 mL sodium phosphate buffer (20 mM, pH 6.5) were added. The solution was put in a boiling water bath for 15 min. After cooling to room temperature, 0.2 mL lichenase (10 U) was added, and the solution was incubated for 60 min at 50 °C. Sodium acetate buffer (5.0 mL, 200 mM, pH 4.0) was added, the tubes were vortexed and centrifuged (1000× *g*, 10 min, 20 °C). The supernatant (0.1 mL) was transferred to a test tube in duplicate. To the first test tube, 0.10 mL of β-glucosidase (0.2 U) was added, while 0.10 mL sodium acetate buffer was added to the second test tube. After incubation for 10 min at 50 °C, 3.0 mL of glucose oxidase/peroxidase reagents was added, and after 20 min, absorbance was measured at 510 nm. The WE-β-glucan content was calculated, as explained in the Megazyme procedure. Fructan contents were analysed as described in Verspreet et al. [5]. WE-protein content was measured using the Dumas combustion method with an automated protein analysis system (factor 6.25) (Vario MAX CN, Elt, Gouda, Netherland). All analyses were conducted in triplicate.

### 2.7. Analysis of In Vitro Fermentation Characteristics

An in vitro fermentation experiment was performed to evaluate the effect of extrusion-cooking on the in vitro fermentation characteristics of wheat bran. It was decided to exclude the predigestion step before the in vitro fermentation experiment, as Karpinnen, Myllymäki, Forssell and Poutanen [33] showed that fructan, and possibly also WE-AX, is removed during in vitro predigestion. Removal of these components prevents proper evaluation of the fermentation characteristics. The sample that was modified the most and had the highest WE-AX content was selected, i.e., VHS23. A blank, without the addition of bran, was included to correct for SCFA production from fermentable substrates in the pooled faecal slurry. The control sample that has the same particle size as the extruded samples and underwent the same heat treatment during drying as the extruded samples was included in the experiment as reference.

Preparation of the pooled human faecal slurry (10% *w*/*v*) was done as described in Roye et al. [24]. For each sample, an amount of sample containing 100.0 mg of dietary fibre was weighed (accounting for starch, protein, lipid, phytate and ash content). An amount of 25.0 mL of faecal slurry was added, the headspace was flushed with nitrogen gas, and the tubes were firmly closed. Incubation was at 37 °C for 2, 4, 8, 24 and 48 h. pH and SCFA concentration (acetate, propionate, butyrate, isobutyrate and isovalerate) were determined at every time point as described in Roye et al. [24].

### 2.8. Statistical Analysis

Statistical data treatment was performed using R and R studio software (version 3.5.2). The dataset consisted of 16 observations (*n* = 16), distributed among 3 controlled factors of varying levels: configuration (low, medium, high and very high shear), total moisture content during extrusion-cooking (23, 27 and 33%) and set temperature in the last barrel (120 and 145 °C). Two types of output variables (Y) were measured: 3 extrusion dependent outputs (outlet pressure, product temperature and SME) and 5 analytically measured outputs (SWBC, extractability, extract viscosity, WE-AX and arabinose to xylose (A/X) ratio). Importance of controlled factors on all variables was evaluated through linear regression. As the data set was not balanced (i.e., a differing number of observations per level of each factor), the correlation between controlled factors was first checked. The maximum correlation found did not exceed 11%, hence the order of the terms in the model was kept constant. Linear models were written according to the following equation: Y = α + β_1_ × Configuration + β_2_ × Temperature + β_3_ × Moisture.

Analysis of Variance (ANOVA) tables from these models were extracted, and the significance of extrusion-cooking parameters on each variable was determined through *p*-values with a significance threshold set at 5%. In case of a significant impact of terms, goodness of linear regression was further determined using predicted versus observed plots and adjusted R^2^ derived from the model. The specific impact of SME on analytically measured outputs was further evaluated through the use of simple linear regression (Y = α + β × SME). The importance of SME was evaluated using the *p*-values obtained from the above linear regression and by building the corresponding scatterplots. Goodness of modelling was evaluated with an adjusted R^2^ for every model.

## 3. Results and Discussion

### 3.1. Modelling of the Effect of Screw Configuration, Moisture Content and Last-Barrel Set Temperature on Process Parameters

Sixteen (16) samples were produced with varying screw configuration, moisture content and barrel temperature profile (Table 1). The study showed that a stable production process could only be obtained if the total moisture content during wheat bran extrusion-cooking was 23% or higher and if the last-barrel set temperature was not higher than 145 °C. Moreover, the screw configuration was changed to increase shear stepwise, within the limitations of the screw elements available.

**Figure 2:**
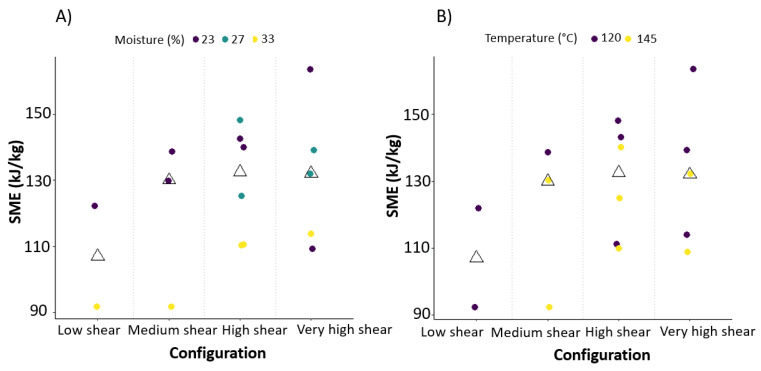
Scatterplot of the specific mechanical energy (SME) according to screw configuration. The triangles represent the median obtained by configuration. (**A**) Colouring of data points according to moisture in the extruder. (**B**) Colouring of data points according to temperature applied in the last barrel.

All three extrusion-dependent variables (outlet pressure, product temperature, SME) were significantly impacted by extrusion parameters, in a more or less pronounced fashion (see measured values in Table 1 and *p*-values in Table 2). Barrel set temperature and moisture content significantly influenced outlet pressure, product temperature and SME, as previously described in the literature [20,34,35]. SME values between 92 kJ/kg and 164 kJ/kg were measured with the highest value reached for VHS23. The lowest SME was found for LS33. Outlet pressure in the extruder reached values between 22 and 58 Bar. Outlet pressure was higher when the total moisture content was low and when the last-barrel set temperature was low. As expected, increasing the barrel temperatures increased product temperature. Product temperature also increased with decreasing moisture content, likely due to more friction between bran particles themselves and between bran and extruder, leading to heat generation. These effects could be fitted with good accuracy (adjusted R^2^ > 0.77) (see observed versus predicted plots in Figure S1). These results can constitute a prediction tool that can prove useful when running supplementary trials if, for example, a defined SME or product temperature needs to be achieved. Interestingly, changing the screw configuration resulted in highly significant changes in product temperature (*p* < 0.0001) and in a less significant yet visible trend on SME (0.1 > *p* > 0.05). Figure 2A,B illustrate these effects. As shown in Figure 2, SME increased when more work sections were introduced in the screw configuration. Decreasing the moisture content increased SME (Figure 2A) due to increased friction. A previous study [20] observed as well that SME increased with decreasing moisture content. In addition, a low last-barrel set temperature resulted in higher SME values compared to high temperatures in the last barrel (Figure 2B).

### 3.2. Effect of Extrusion-Cooking on the Structure of Wheat Bran

**Figure 3:**
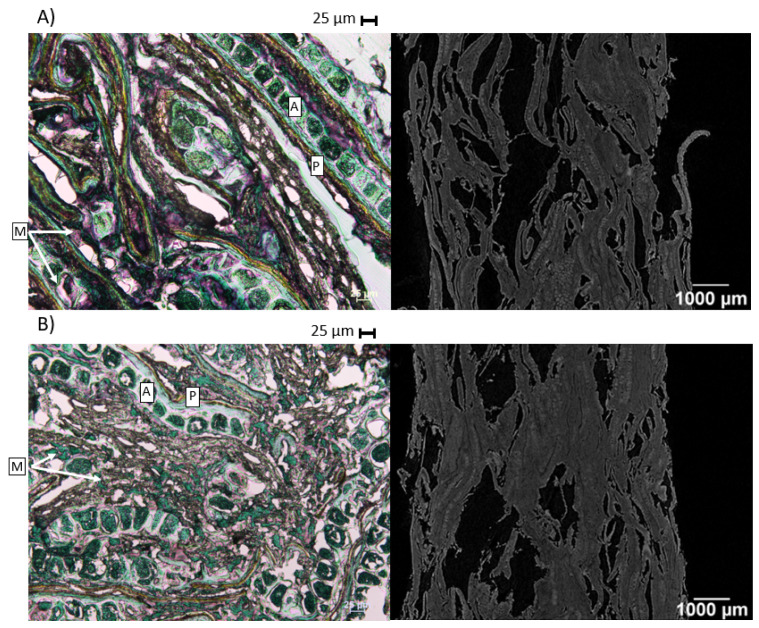
Light microscopy pictures (**left**), where starch is stained with Lugol solution (purple) and proteins are stained with light green (green) and X-ray tomography pictures (**right**) of (**A**) low shear extruded wheat bran at 23% of total moisture using the low-temperature profile and (**B**) very high shear extruded wheat bran at 23% of total moisture using the low-temperature profile. P = pericarp; A = aleurone; M = matrix surrounding the bran particles.

The structure of two samples (LS23 and VHS23) after exiting the extruder, but prior to milling, was visualised (Figure 3), to evaluate the effects of LS and VHS extrusion-cooking on the product macrostructure and bran structure. With X-ray tomography, it was seen that both products incorporated air in their macrostructure, visible as open spaces inbetween the bran particles. Nevertheless, these voids are much smaller compared to other extruded products like rice products and extruded starchy foams [26,36]. This is probably partly due to the low overall starch content of the bran material. At least 10% to 25% of starch is needed to induce enough viscosity to result in flow of extrusion melt. At the lower limit of this range, which is the case for the wheat bran we used (12% of starch), the spaces between the fibre fragments are not filled [37], and less product expansion will occur. The observation is also consistent with the findings of Robin et al. [36] that the addition of wheat bran to starchy foams reduced expansion. This was ascribed to the effect of bran on the viscoelastic properties of the product in the extruder and the disruption of the continuous structure of the melt [38].

When focusing on the individual wheat bran structures, the porous pericarp structure (±30–40 µm thickness) and the aleurone layer (±50 µm thickness) can still be discerned (Figure 3A,B) in both samples. VHS extrusion-cooking did degrade the pericarp structure and aleurone layer to a larger extent than LS extrusion-cooking. Broken aleurone cells, as well as a disrupted pericarp structure, are visible on the light microscopy pictures. Overall, more work sections in the screw configuration led to more shear and thus more structure breakdown. Moreover, wheat bran pericarp in the VHS23 sample was surrounded by a matrix, which was less the case for the LS23 sample. This matrix possibly consists of starch, proteins and solubilised bran material that acts as a lubricant during extrusion-cooking.

### 3.3. Modelling of the Effect of Screw Configuration, Total Moisture Content and Barrel Temperature on Bran–Water Interaction and Extract Viscosity

**Figure 4:**
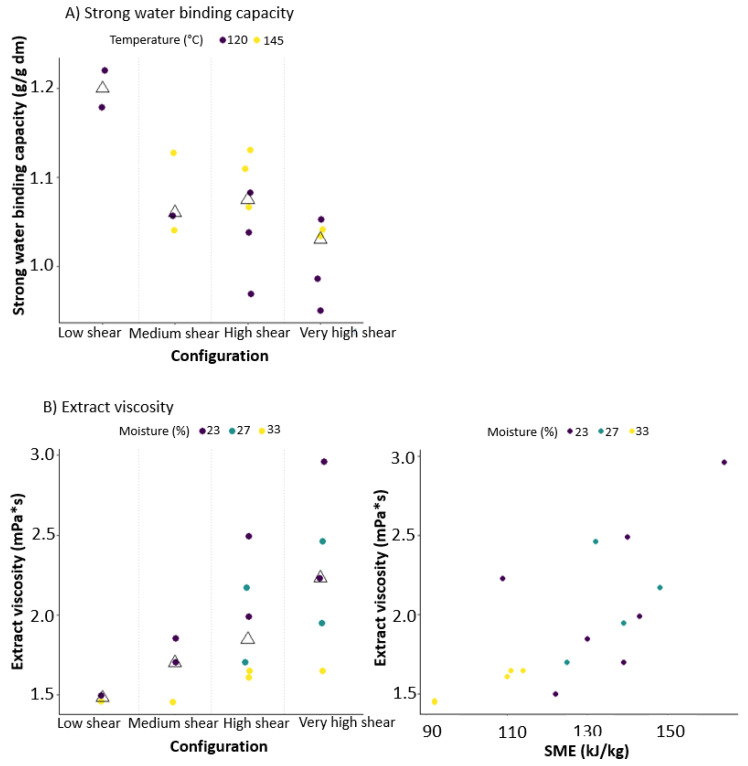
Scatterplot of (**A**) strong water-binding capacity of extruded wheat bran samples according to screw configuration used during processing. Data points are coloured according to last-barrel set temperature and (**B**) (**left**) extract viscosity according to screw configuration and (**right**) according to specific mechanical energy (SME). Data points are coloured according to moisture content during extrusion-cooking The triangles represent the median obtained by configuration.

Extrusion-cooking resulted in an overall increase of the strong water-binding capacity (SWBC) of wheat bran. The control sample (0.88 g/g dm) had a lower SWBC compared to all extruded samples (0.95–1.22 g/g dm). The highest SWBC was measured for LS23 (1.22 g/g dm), while the lowest was measured for HS33 (0.97 g/g dm), VHS27 (0.95 g/g dm) and VHS23 (0.99 g/g dm). Linear modelling highlighted that the screw configuration set-up, and the last-barrel set temperature significantly impacted SWBC (*p* values < 0.05, see Table 2). Figure 4A illustrates both effects, and these effects could be fitted with good accuracy (adjusted R^2^ = 0.76) (see observed versus predicted plots in Figure S2). SWBC decreased when the number of work sections increased in the screw configuration, which can be related to the increase in shear that results in more degradation and disruption of the wheat bran structure, thus decreasing the amount of water that can be bound in nanopores and capillaries. The increased compression, leading to compacting of the overall bran structure, can explain the decreased SWBC as well. Increasing the last-barrel set temperature led to a higher SWBC, probably because a more vigorous flash-off is favoured at higher product temperatures. Decreasing the moisture content showed a tendency to increase SWBC, possibly due to the higher pressures reached and thus a more pronounced pressure drop. The increase in SWBC as a result of extrusion-cooking is consistent with the results of Yan, Ye and Chen [21] for wheat bran and Gutkoski and El-Dash [39] for oat bran, although the measurement methods used are different. The increase in SWBC can be explained by the high pressure and temperature in the extruder, followed by the pressure drop when extruded wheat bran exits the extruder at the die. Water that is present in the pores of bran will evaporate forcefully and cause expansion [16,40]. If the severity of the treatment and thus SME is increased, a decrease in water-binding capacity is observed. The same conclusions were drawn by Ralet, Della Valle and Thibault [41] for pea hulls.

Extract viscosity was measured as an indication for structure breakdown and solubilisation of molecules. Extrusion-cooking resulted in an increased extract viscosity for all samples (from 1.3 mPa·s to 1.5–3.0 mPa·s) (Table 1) due to structure degradation by shear. The highest extract viscosity was measured for VHS23, while the lowest was measured for LS33. An increasing trend in viscosity when the number of work sections was increased was also observed with linear modelling, showing that screw configuration and moisture content had a tendency to influence the extract viscosity (*p*-values between 0.05 and 0.1, Table 2). This was visualised in Figure 4B. The accuracy of the models is visualised in Figure S2 (adjusted R^2^ = 0.46). The increase in extract viscosity when the moisture content is decreased and the amount of work sections is increased indicates that extract viscosity is dependent on SME, which was shown using linear regression (*p*-values in Table 3) and was visualised in Figure 4B (right) (adjusted R^2^ = 0.50). Extract viscosity clearly increases if SME is increased. Increasing the number of work sections and decreasing the moisture content can thus improve structure degradation and fragmentation. The extent of this increase will depend on the concentration of extractable compounds, as well as the characteristics of these compounds like DP and substitution degree [42]. This is discussed further in Section 3.5. The results obtained in this study can be used to steer wheat bran characteristics by varying process parameters. They can also be used as a basis for further research about wheat bran extrusion-cooking.

### 3.4. Modelling of the Effect of Extrusion-Cooking on a Selection of Chemical Characteristics of Wheat Bran

**Figure 5:**
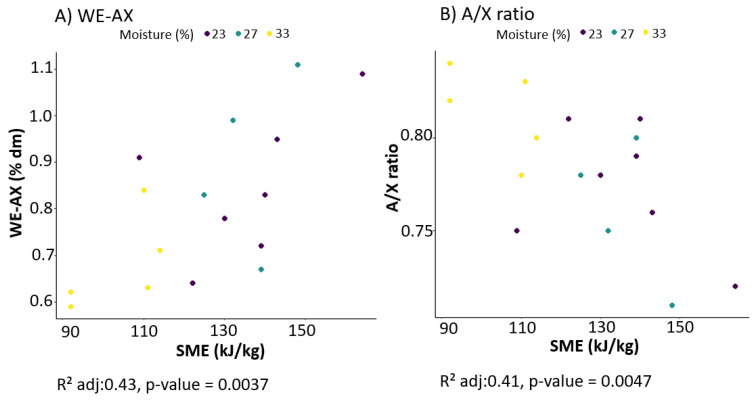
Scatterplot of (**A**) water-extractable arabinoxylan content (WE-AX) and (**B**) arabinose to xylose (A/X) ratio according to specific mechanical energy (SME). Data points are coloured according to moisture content during extrusion-cooking. Adjusted R^2^ and *p*-values were obtained using the simple linear regression of WE-AX and A/X ratio as a function of SME.

As displayed in Table 2, changing the extrusion-cooking parameters we controlled did not per se lead to a significant change in the WE-AX content and A/X ratio. The impact of SME was thus further evaluated through linear regression (Table 3). SME showed clear contribution on both the amount and branching of the AX material extractable from the extruded bran (Figure 5). The accuracy of the models is reflected in the adjusted R^2^ (adjusted R^2^ = 0.43 for WE-AX content and adjusted R^2^ = 0.41 for A/X ratio).

The highest WE-AX content and lowest A/X ratio were observed for VHS23 (1.1% dm WE-AX and A/X ratio of 0.72) and VHS27 (1.1% dm WE-AX and A/X ratio of 0.71). These samples underwent the highest SME (148 kJ/kg and 164 kJ/kg, respectively). The positive correlation between WE-AX and SME is visualised in Figure 5A and shows that the WE-AX concentration is increased if SME is increased. A high moisture content (coloured yellow in Figure 5A) resulted in low SME values and thus a low WE-AX content. For the A/X ratio, a decreasing trend was observed when SME was increased. High A/X ratios were measured for the samples that underwent the lowest SME. The AX in the aleurone of wheat bran are less substituted than in wheat pericarp, as reflected by a lower A/X value (±0.4 versus ±1.1) [43]. It can be hypothesised that mainly WU-AX from the aleurone is thus solubilised due to extrusion-cooking, resulting in a lowered A/X ratio. This suggested the degradation of the aleurone during extrusion-cooking and was also confirmed with light microscopy (see Figure 3). The SME thus showed to be the determining factor for the WE-AX content and A/X ratio. The increase in WE-AX content due to extrusion-cooking was also reported by Andersson et al. [22]. They observed that the lowest moisture content during extrusion-cooking resulted in the highest WE-AX content. Finally, the link between WE-AX content and characteristics on the one hand, and extract viscosity, on the other hand, was evaluated. A linear relation was found between extract viscosity and WE-AX content (plot in Figure S3), which is consistent with the knowledge that WE-AX can induce viscosity in solution [44]. In this study, differences between A/X ratios are possibly too small to induce differences in viscosity, or the effect of concentration is dominating the impact of A/X on viscosity.

### 3.5. In-Depth Analysis of the Effect of Extrusion-Cooking on the Chemical Composition of Wheat Bran

The measured wheat bran characteristics (Table 1) and derived linear models showed that VHS23 had the highest extract viscosity and WE-AX content, and the lowest A/X ratio. This sample was thus degraded the most. The SWBC of this sample was also increased compared to the control sample, showing structure loosening. This sample was, therefore, one of four samples selected for further analysis. Moisture content during extrusion-cooking and screw configuration showed to be determining factors for SME, outlet pressure and product temperature and thereby also extract viscosity and SWBC. To evaluate the effect of moisture content during extrusion-cooking, VHS27 and VHS33 were also selected for further analysis. Finally, LS23 was also selected to evaluate the effect of screw configuration. The samples that were selected for in-depth analysis were hence LS23, VHS23, VHS27 and VHS33.

#### 3.5.1. Dietary Fibre Components

The WE-AX content (Table 4) of VHS23 was the highest (1.09% dm) compared to LS23 (0.64% dm), VHS27 (0.67% dm) and VHS33 (0.70% dm). The A/X ratio of VHS23 (0.72) was also the lowest of the four selected extruded samples.

In addition to WE-AX content and A/X ratio, the average DP of WE-AX was determined. The DP of WE-AX of VHS23 (112) was the highest, showing that the WE-AX fragments of the VHS23 samples were larger compared to the other three samples. This sample also had a high WE-AX content (1.09% dm) and the lowest A/X ratio (0.72). Comparing the samples with the lowest and highest SME treatment highlighted that extrusion-cooking with increased energy enabled more solubilisation of WU-AX into larger fragments that were less substituted. The differences in average DP of WE-AX can also contribute to the effect on extract viscosity, as a high DP leads to a higher viscosity [42,45,46].

For fructans, a rapidly fermentable dietary fibre component [47], no degradation due to extrusion-cooking was observed for LS23, VHS23, VHS27 and VHS33 (Table 4) and no formation of free fructose was detected. Fructan content and structure were thus preserved. Fructan can act as a substrate for fermentation and hence have a prebiotic effect in the colon [48].

Extrusion-cooking resulted in a decrease in WE β-glucan content from 0.37% dm in the control sample to 0.16% dm for LS23, 0.27% dm for VSH23, 0.21% dm for VHS27 and 0.19% dm for VHS33 (Table 4). Extrusion-cooking thus decreased β-glucan extractability to a limited extent. This can be due to the induction of interactions between β-glucan and other bran components like AX and cellulose, as was suggested by Izydorczyk et al. [49]. Interactions with proteins and starch or matrix effects of starch could also be the reason for the decreased extractability [50].

#### 3.5.2. Starch Characteristics

With regard to the characteristics of starch after extrusion-cooking, it was seen that Maltese crosses were absent on polarised light microscopic pictures (results not shown). Treating extruded wheat bran with α-amylase and amyloglucosidase showed that all starch (12% dm) could be enzymatically degraded within a short amount of time. This shows that all starch was melted or damaged during extrusion-cooking, corresponding with results in the literature [16]. It is moreover known that extrusion-cooking is one of the only processes in which starch melts at low moisture contents (10–34%), due to the high temperatures applied and due to friction occurring in the extruder [51].

Extrusion-cooking also led to starch solubilisation, as the content of WE polymeric glucose increased from 3.72% dm to 5.93 dm%–7.09 dm% (Table 4). Additionally, it was shown that the DP of these extractable fragments was higher (37–63) compared to the WE fragments in the control sample (11). The highest WE polymeric glucose content (7.01% dm) and lowest DP value (38) amongst the 4 selected samples was found for VHS23, the sample for which the highest SME was reached. Liu, Halley and Gilbert [52] also showed that extrusion-cooking of starch led to starch melting and starch depolymerisation. They moreover showed that the degradation mechanism preferentially operates on large molecules, leading to a narrowing of the size distribution. Chanvrier et al. [26] showed a decrease in the molecular weight of starch, and linked the extent of this decrease to an increase in SME. The results of the current study show an increase in WE polymeric glucose content and a decrease in average DP when SME is increased, and this can be attributed to more starch depolymerisation. Despite the degradation of starch, no increase in free glucose was found as a result of extrusion-cooking.

#### 3.5.3. Extractable Protein Content

The extractable protein content decreased during extrusion-cooking from 5.8% dm to 1.4–1.7% dm. This result is in alignment with the heat-induced protein denaturation described in Rombouts, Lagrain and Delcour [53]. Several other extrusion-cooking studies also showed a loss in protein solubility as a result of protein polymerisation through the creation of disulphide bonds [26,54] and non-covalent molecular interactions with other proteins or dietary fibre [55,56].

#### 3.5.4. Phytate and Free Ferulic Acid

Phytate content in the selected extruded samples is presented in Table 4. The control sample contained 4.3% dm of phytate, while the extruded samples had a somewhat lower phytate concentration (3.7% dm for LS23, 3.5% dm for VHS23, 3.9% dm for VHS27 and 4.1% dm for VHS33). This shows that extrusion-cooking resulted in partial phytate breakdown, with a decrease in phytate content of 18.6% dm for VHS23. The mechanism behind this phytate breakdown can be assumed to be enzymatic [57], thermal [58] and/or due to shear. Enzymes are nevertheless inactivated after the extrusion-cooking treatment (results not shown), indicating enzymatic degradation only at the start of the extrusion-cooking process.

Extrusion-cooking also led to a substantial increase in free ferulic acid content from 7.1 mg/kg dm to 16.5–25.1 mg/kg dm. The sample that underwent the highest SME also showed the highest release of ferulic acid, as free ferulic acid content was increased with a factor of 3.5 for VHS23. Ferulic acid is for its major part bound to arabinose in AX by an ester bond. The release of free ferulic acid could reflect breakage of this ester bond and can thus be used as a measure of molecular structure degradation [59]. The release of ferulic acid due to extrusion-cooking of wheat bran was also shown in a previous study [20]. The extent of the increase depended on the feed rate of wheat bran (tested for 20–32 kg/h) and moisture content (tested for 6.1–17.5% of dry solid feed rate). The screw configuration was, however, not taken into account. Our study clearly shows that increasing shear by increasing the number of work sections in the screw configuration leads to the release of ferulic acid.

### 3.6. Effect of Extruded Wheat Bran Properties on In Vitro Fermentation Characteristics

**Figure 6:**
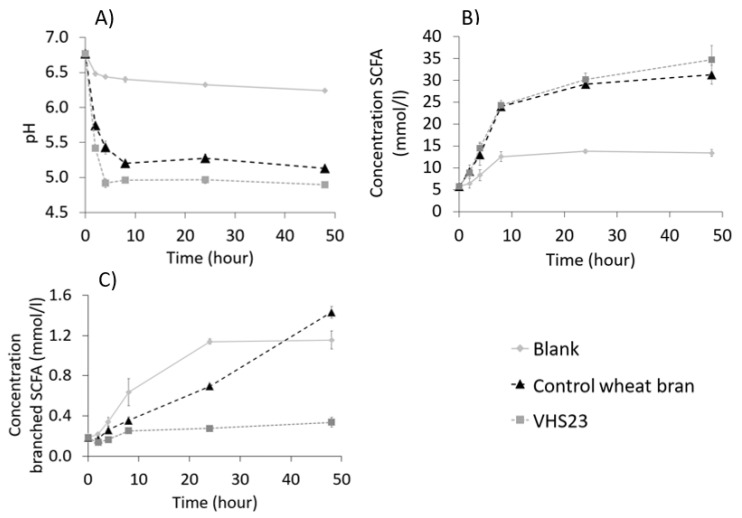
(**A**) pH, (**B**) concentration of short-chain fatty acids (SCFA) (sum of acetate, propionate, butyrate, isobutyrate, isovalerate; mmol/L) and (**C**) concentration of branched SCFA (isobutyrate and isovalerate, mmol/L) as a function of time obtained during an in vitro fermentation experiment in a 10% faecal slurry of a blank sample (no bran addition), control wheat bran and very high shear extruded wheat bran at 23% of moisture (VHS23).

An in vitro fermentation experiment was conducted, which was not preceded by in vitro predigestion, as elaborated in Section 2.7. The effect of extrusion-cooking on the fermentation rate and fermentation degree was investigated by following the pH and the SCFA concentration in function of fermentation time during an in vitro fermentation experiment using a pooled faecal slurry (Figure 6). The sample that was most strongly modified (VHS23) was selected for the in vitro fermentation experiment. Control wheat bran was included, as well as a blank sample with no addition of bran. The pH over time was always lower for both control and VHS23 compared to the blank (Figure 6A), and SCFA concentration was consequently always higher than for the blank (Figure 6B). This shows fermentation of both samples. The pH of the blank decreased from 6.7 at the beginning of fermentation to 6.2 after 48 h of faecal fermentation. This is explained by the possible presence of some residual dietary fibre in the faecal slurry. The pH for VHS23 dropped the fastest; the pH was 5.4 after 2 h of fermentation and 4.9 after 4 h, while it was respectively 5.7 and 5.4 for the control wheat bran. After 48 h, the pH of the control sample was 5.1 and the pH of extruded wheat bran was 4.9. SCFA concentration after 2 and 4 h of fermentation was the same for both control and VHS23 (Figure 6B). However, after 48 h of fermentation, SCFA concentration was slightly higher for VHS23 (35 mmol/L) than for the control wheat bran (31 mmol/L). The behaviour of the control and VHS23 upon fermentation hence looked very similar, with a slightly higher fermentation rate and degree for VHS23.

As presented in the starch characterisation paragraph, starch was melted due to extrusion-cooking, while native starch granules are present in the control sample. It can be expected that melted starch will be fermented faster compared to native starch, leading to differences in fermentation kinetics that are not related to dietary fibre fermentation. Previous studies, however, have shown that complete fermentation of wheat starch granules in vitro or in vivo typically happens within 24 h [60,61]. This would imply that at 48 h, the difference in SCFA concentration and pH between the control and VHS23 can be attributed to the changes that wheat bran underwent during extrusion-cooking. After 48 h, it was seen that extrusion-cooking led to a slightly lower pH and higher SCFA production compared to the control. This increase in fermentation degree due to extrusion-cooking can be attributed to the more accessible structure and the changed chemical composition. The fructan levels in the extract are similar between the control bran and VHS23 (see Table 4). The slight changes can, therefore, be attributed to the increase in WE-AX content from 0.62% dm for the control to 1.09% dm for VHS23 (Table 4). This increase could kick-start the microbiota at the beginning of fermentation, resulting in a more efficient fermentation at later fermentation stages, e.g., at 48 h. Moreover, an increased SWBC indicates that the bran structure is more accessible for the microbiota, thus allowing slightly higher fermentation, in accordance to the hypothesis of Bäckhed and coworkers [62] and Roye and coworkers [24]. In conclusion, the results show that the studied extrusion-cooking conditions induced changes in the physicochemical characteristics of bran which were not sufficiently large to have a drastic effect on the extent of saccharolytic fermentation in 48 h.

Changes in the production of branched SCFA as a function of time due to extrusion-cooking were observed. Less branched SCFA were produced for extruded wheat bran compared to control wheat bran (Figure 6C): after 48 h of fermentation, the concentration of branched SCFA was 1.4 mmol/L for the control wheat bran, compared to 0.3 mmol/L for VHS23. It can be hypothesised that the microorganism did not shift their metabolism for energy production to protein fermentation as the accessible structure and increased WE-AX content that could kick-start the microbiota led to a better carbohydrate metabolisation. In addition, the formation of non-peptide bonds between proteins and other bran compounds can also be hypothesised to be part of the explanation.

### 3.7. Overview of the Results

Different screw configurations, barrel temperature profiles and moisture contents were tested using a pilot-scale BC21 extruder, aiming for the highest shear possible but ensuring a stable extrusion-cooking process.

Extrusion-cooking increased the SWBC of wheat bran, and this effect was most outspoken when decreasing the moisture content and increasing last-barrel set temperature for all screw configurations tested. Low moisture and high-temperature conditions possibly led to a stronger water flash-off at the end of the die, increasing SWBC due to a more pronounced expansion. An increase in water-binding capacity of bran could lead to an increase in faecal bulk and normalisation of transit time [63]. Clinical studies would be needed to establish this effect in vivo with extruded bran. When more work sections were used in the screw configuration, SWBC was decreased due to structure breakdown, also resulting in an increase in extract viscosity. Extract viscosity could moreover be increased by decreasing the moisture content during extrusion-cooking. The extract viscosity increase was attributed to the increase in extractable compounds such as WE-AX and extractable polymeric glucose, which was most evident for the samples submitted to the highest mechanical shear. The increase in DP of WE-AX also contributes to the increased extract viscosity. Moreover, WE-AX solubilisation seems to mainly take place in the aleurone. This led to the assumption that aleurone cells are partially opened, thus increasing the availability of bioactive compounds. Despite the high shear applied and clear solubilisation of AX, no fructan degradation was seen as a result of extrusion-cooking, allowing fructan to exert its prebiotic effect [5]. Moreover, phytate was partly degraded during extrusion-cooking, and most so for VHS23. This may lead to increased mineral bioaccessibility. The highest shear also led to the highest increase in free ferulic acid, also showing more evidence of the degradation of the wheat bran structure. It could be hypothesised that solubilisation of ferulic acid can increase its absorption in the small intestine, allowing it to act as antioxidant [15]. Nevertheless, it can be questioned if the increase in free ferulic acid is large enough to induce this effect.

For the faecal fermentation characteristics, extrusion-cooking of wheat bran under the conditions reported in this work only resulted in a small pH decrease and a small increase in SCFA production after 48 h of fermentation. It was therefore concluded that the fermentation degree was increased only to a limited extent. Despite the limited effect on carbohydrate fermentation characteristics, protein fermentation was lower for the extruded wheat bran sample in comparison to the control. This could imply that less potentially toxic compounds like phenols and ammonia would be produced if extruded wheat bran is incorporated into food products [64].

## 4. Conclusions

The potential of extrusion-cooking as a modification strategy to change the physicochemical characteristics of wheat bran and thereby improve the nutrition-related properties was explored. The importance of screw configuration, moisture content and last-barrel set temperature on the extent of this effect was evaluated. It was the first time, to the best of our knowledge, that screw configuration was taken into account in such a study.

Moisture content and screw configuration are important for SWBC and extract viscosity, while the last-barrel set temperature only determined SWBC. The effect of screw configuration was more pronounced than the effect of moisture content. Both effects were mainly attributed to changes in SME. More structure degradation was seen if higher SMEs were reached. The particular combination of a screw configuration with a high amount of work sections and a low moisture content during extrusion-cooking led to the most substantial structure degradation. The extent of this degradation could not be reached by using a screw configuration with a lower amount of work sections or by using the very high shear screw configuration at higher moisture contents. This shows that the combination of the very high shear screw configuration and low moisture content is key to degrade the structure as much as possible. The highest WE-AX levels were reached, the highest phytate degradation occurred, most ferulic acid was released and aleurone cells were shown to be broken down using this particular set-up. This could lead to an increased bioaccessibility of bioactives and a higher antioxidant capacity. The fermentation rate was, however, only increased to a limited extent.

While bran modifications were smaller than could be expected, given the high energy input, the knowledge on how wheat bran characteristics can be modified to a certain extent by changing moisture content, screw configuration and last-barrel set temperature can be used for goal-oriented modifications. The implementation of modified wheat bran in end products can be based on the intended physiological effects or on the food product used. However, clinical studies would be needed to gain more information about the added value of extrusion-cooking of wheat bran on health. In addition, the effect of extrusion-cooking on techno-functional properties of wheat bran should also be evaluated. One could, for example, hypothesise that the expansion of the wheat bran structure could impact texture and mouthfeel properties when added to food products.

## Figures and Tables

**Table 1:** Process parameters and product properties of the 16 samples obtained by extrusion-cooking with different screw configurations, different last-barrel set temperatures and different moisture contents. The four selected samples for in-depth analysis are underlined. The data represent a single extrusion-cooking experiment starting from one batch of wheat bran. Standard deviations are indicated between brackets. SME—specific mechanical energy, SWBC—strong water-binding capacity.

Configuration	Moisture Content (%)	Screw Speed (rpm)	Temperature in the Last Barrel (°C)	Outlet Pressure (Bar)	SME (kJ/kg)	Product Temperature (°C)	SWBC (g/g dm)	Extract Viscosity (mPa·s)	Sample Coding
Low shear	33	310	120	25.6	92	105.1	1.18 (±0.03)	1.46 (±0.03)	LS33
Low shear	23	310	120	39.6	122	114.1	1.22 (±0.01)	1.50 (±0.00)	LS23
Medium shear	23	310	120	58.4	139	121.7	1.06 (±0.04)	1.7 (±0.03)	MS23
Medium shear	33	310	145	21.6	92	129.5	1.04 (±0.08)	1.45 (±0.03)	MS33T
Medium shear	23	310	145	41.7	130	139.7	1.13 (±0.14)	1.85 (±0.02)	MS23T
High shear	27	310	120	60.8	148	123.9	1.08 (±0.04)	2.17 (±0.05)	HS27
High shear	23	310	120	42.7	143	118.5	1.04 (±0.01)	1.99 (±0.04)	HS23
High shear	33	310	120	22.0	111	105.6	0.97 (±0.04)	1.65 (±0.02)	HS33
High shear	33	310	145	18.1	110	123.8	1.11 (±0.04)	1.61 (±0.04)	HS33T
High shear	27	310	145	28.2	125	129.6	1.07 (±0.02)	1.70 (±0.04)	HS27T
High shear	23	310	145	36.7	140	129.2	1.13 (±0.08)	2.49 (±0.05)	HS23T
Very high shear	33	310	120	27.2	114	106.8	0.99 (±0.04)	1.65 (±0.1)	VHS33
Very high shear	27	310	120	45.1	139	113.8	0.95 (±0.03)	1.95 (±0.03)	VHS27
Very high shear	23	310	120	52.9	164	120.6	1.05 (±0.03)	2.96 (±0.03)	VHS23
Very high shear	27	310	145	37.3	132	129.8	1.03 (±0.03)	2.46 (±0.02)	VHS27T
Very high shear	23	310	145	37.8	109	133.5	1.04 (±0.01)	2.23 (±0.02)	VHS23T

**Table 2:** *p*-value table of the linear model describing the effect of the model terms (screw configuration, last-barrel set temperature and moisture content) on the extrusion dependent variables (outlet pressure, specific mechanical energy (SME), product temperature at the extruder outlet) and the measured product variables (strong water binding capacity (SWBC), extractability, extract viscosity, water-extractable arabinoxylan (WE-AX) content and arabinose to xylose (A/X) ratio). The model was built for each output variables (Y) using the following equation: Y = α + β_1_ × Configuration + β_2_ × Temperature + β_3_ × Moisture. The output variable is determined by the model terms if *p*-value is smaller than 0.05 (significance threshold).

Model Terms (Controlled Factors)			
Output Variables	Screw Configuration	Temperature (Last Barrel)	Moisture Content
Extrusion-dependent variables			
Outlet Pressure	0.3252	0.0038	0.0006
SME	0.0907	0.0234	0.0023
Product Temperature	0.0006	<0.0001	0.0014
Measured product variables			
SWBC	0.0012	0.0275	0.0963
Extractability	0.1452	0.6705	0.6033
Extract Viscosity	0.0543	0.8002	0.0613
WE-AX content	0.1327	0.8705	0.1685
A/X ratio	0.2125	0.8273	0.1602

**Table 3:** *p*-value table of the linear model describing the effect of specific mechanical energy (SME) on the measured variables (strong water-binding capacity (SWBC), extract viscosity, water-extractable arabinoxylan (WE-AX) content and arabinose to xylose (A/X) ratio). The model was built for each output variables (Y) using the following equation: Y = α + β × SME. The output variable is determined by SME if the *p*-value is smaller than 0.05 (significance threshold).

Output Variables	SME
Measured variables	
SWBC	0.6241
Extract Viscosity	0.0013
WE-AX	0.0037
A/X ratio	0.0047

**Table 4:** Water-extractable arabinoxylan content (WE-AX), average degree of polymerisation (DP) of WE-AX, arabinose to xylose ratio (A/X), water-extractable (WE)β-glucan content, fructan content, WE polymeric glucose content, DP of WE polymer glucose, WE protein content, free sugar content (arabinose, xylose, fructose, glucose), free ferulic acid content and phytate content for the different samples: Control, LS23, VHS23, VHS27, VHS33. The data represent the means of triplicate measurements of one extrusion-cooking experiment. Standard deviations are indicated between brackets.

	Control	LS23	VHS23	VHS27	VHS33
WE-AX					
Concentration (% dm)	0.62 (±0.01)	0.64 (±0.01)	1.09 (±0.01)	0.67 (±0.05)	0.70 (±0.03)
DP	45 (±1)	58 (±1)	112 (±1)	63 (±6)	57 (±2)
A/X	0.88 (±0.00)	0.81 (±0.01)	0.72 (±0.01)	0.80 (±0.01)	0.80 (±0.01)
WE-β-glucan (% dm)	0.37 (±0.01)	0.16 (±0.01)	0.27 (±0.01)	0.21 (±0.01)	0.19 (±0.01)
Fructan (% dm)	3.69 (±0.03)	3.94 (±0.28)	3.73 (±0.01)	3.63 (±0.20)	3.51 (±0.10)
WE polymeric glucose					
Concentration (% dm)	3.7 (±0.1)	5.93 (±0.07)	7.1 (±0.0)	6.25 (±0.06)	6.33 (±0.11)
DP	11 (±0)	63 (±2)	38 (±1)	46 (±1)	58 (±6)
WE Extractable proteins (% dm)	5.8 (±0.1)	1.4 (±0.0)	1.7 (±0.1)	1.5 (±0.1)	1.5 (±0.0)
WE free sugars (% dm)	0.8 (±0.0)	0.3 (±0.0)	0.9 (±0.0)	0.7 (±0.0)	0.7 (±0.0)
Free ferulic acid (mg/kg dm)	7.1 (±0.8)	19.2 (±1.7)	25.1 (±0.4)	18.6 (±0.7)	16.5 (±0.2)
Phytate content (% dm)	4.3 (±0.0)	3.7 (±0.0)	3.5 (±0.0)	3.9 (±0.0)	4.1 (±0.1)

## Supplementary Materials

The following are available online at https://www.mdpi.com/2304-8158/9/6/738/s1, Figure S1: Observed versus predicted plots and R^2^ adjusted of the linear model build for (**A**) Output pressure, (**B**) Product temperature and (**C**) Specific mechanical energy using the following equation: Y = α + β_1_ × Configuration + β_2_ × Temperature + β_3_ × Moisture; Figure S2: Observed versus predicted plots and adjusted R^2^ of the linear model build for (**A**) strong water-binding capacity and (**B**) extract viscosity using the following equation: Y = α + β_1_ × Configuration + β_2_ × Temperature + β_3_ × Moisture; Figure S3: Plot of extract viscosity as a function of WE-AX content. The plot has an R^2^ of 0.63.

## Abbreviations

A/X ratio	arabinose to xylose ratio
dm	dry matter
DP	degree of polymerisation
HS	high shear
LS	low shear
MS	medium shear
rpm	rotations per minute
SCFA	short-chain fatty acids
SME	specific mechanical energy
SWBC	strong water-binding capacity
VHS	very high shear
WE	water-extractable
WE-AX	water-extractable arabinoxylan
WU-AX	water-unextractable arabinoxylan
