# Supplementary material for: Extrusion-Cooking Modifies Physicochemical and Nutrition-Related Properties of Wheat Bran
Source: Foods. 2020 Jun 4;9(6):738. doi: 10.3390/foods9060738 (PMC7353595; doi:10.3390/foods9060738)
Supplement: Supplementary file 1 [file foods-09-00738-s001.docx]

Appendix A

**
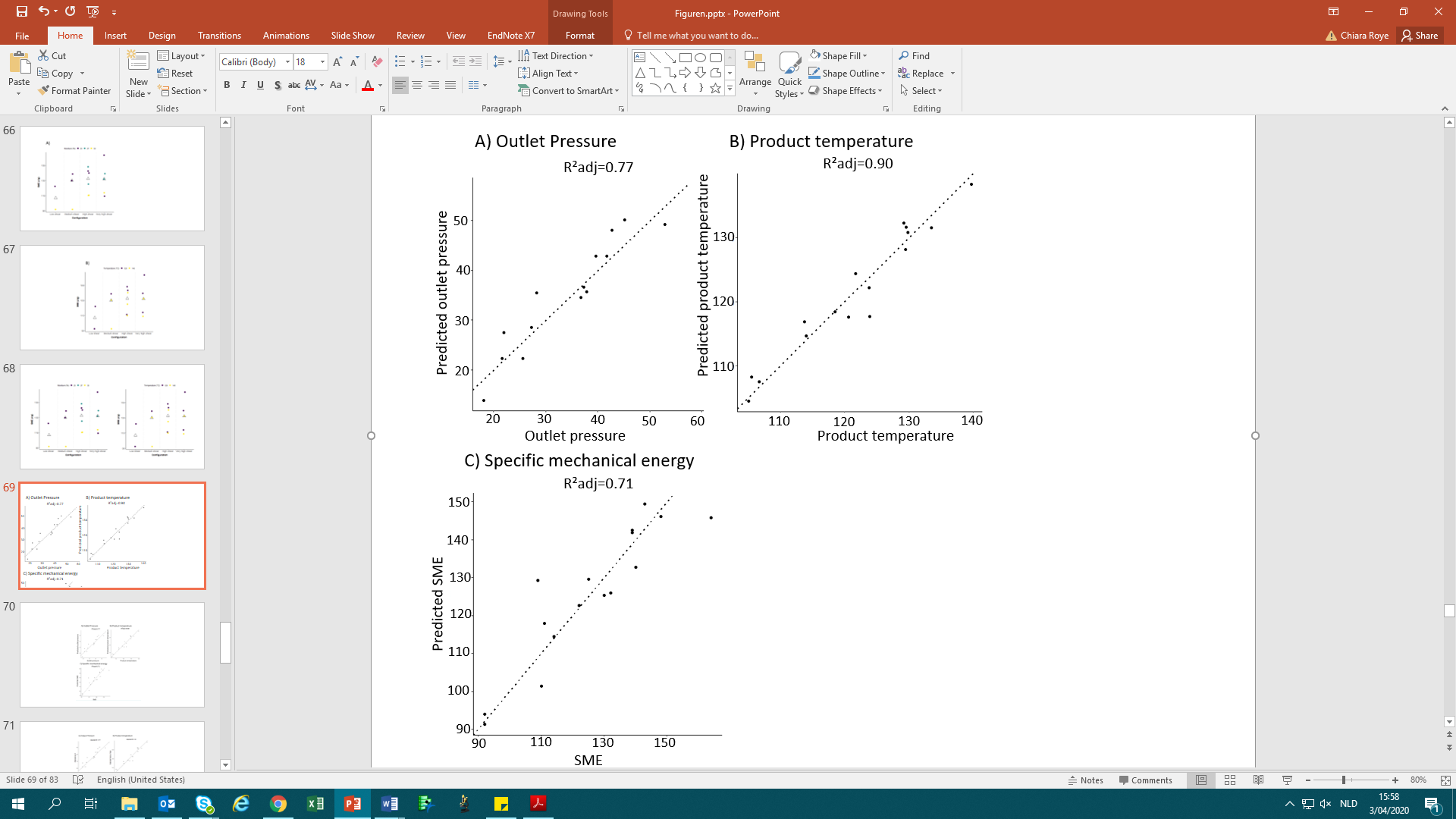
**

**Figure S1**: Observed versus predicted plots and R² adjusted of the linear model build for A) Output pressure, B) Product temperature and C) Specific mechanical energy using the following equation: Y = α + β_1_ * Configuration + β_2_ * Temperature + β_3_ * Moisture


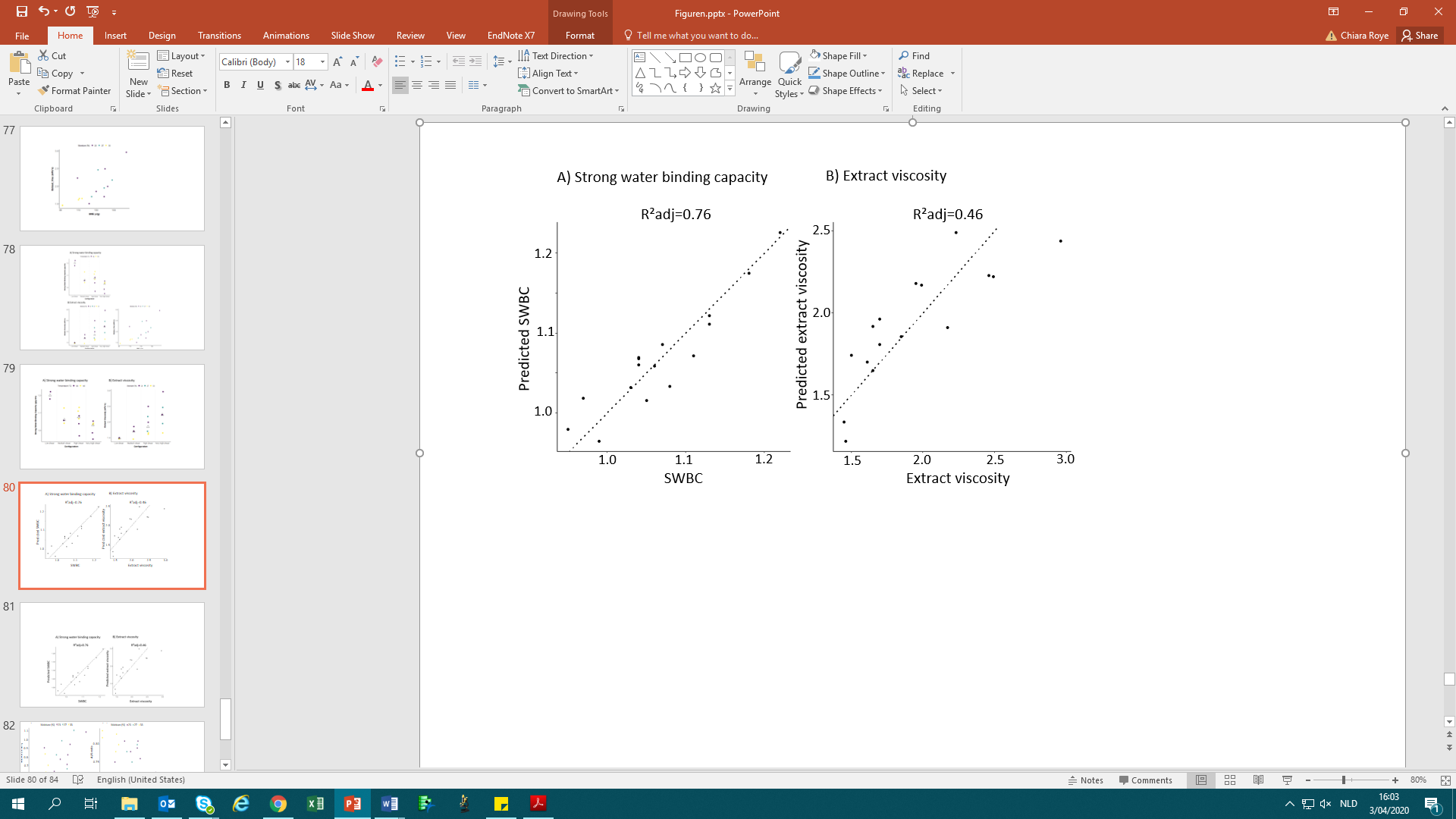


**Figure S2**: Observed versus predicted plots and adjusted R² of the linear model build for A) strong water binding capacity and B) extract viscosity using the following equation: Y = α + β_1_ * Configuration + β_2_ * Temperature + β_3_ * Moisture


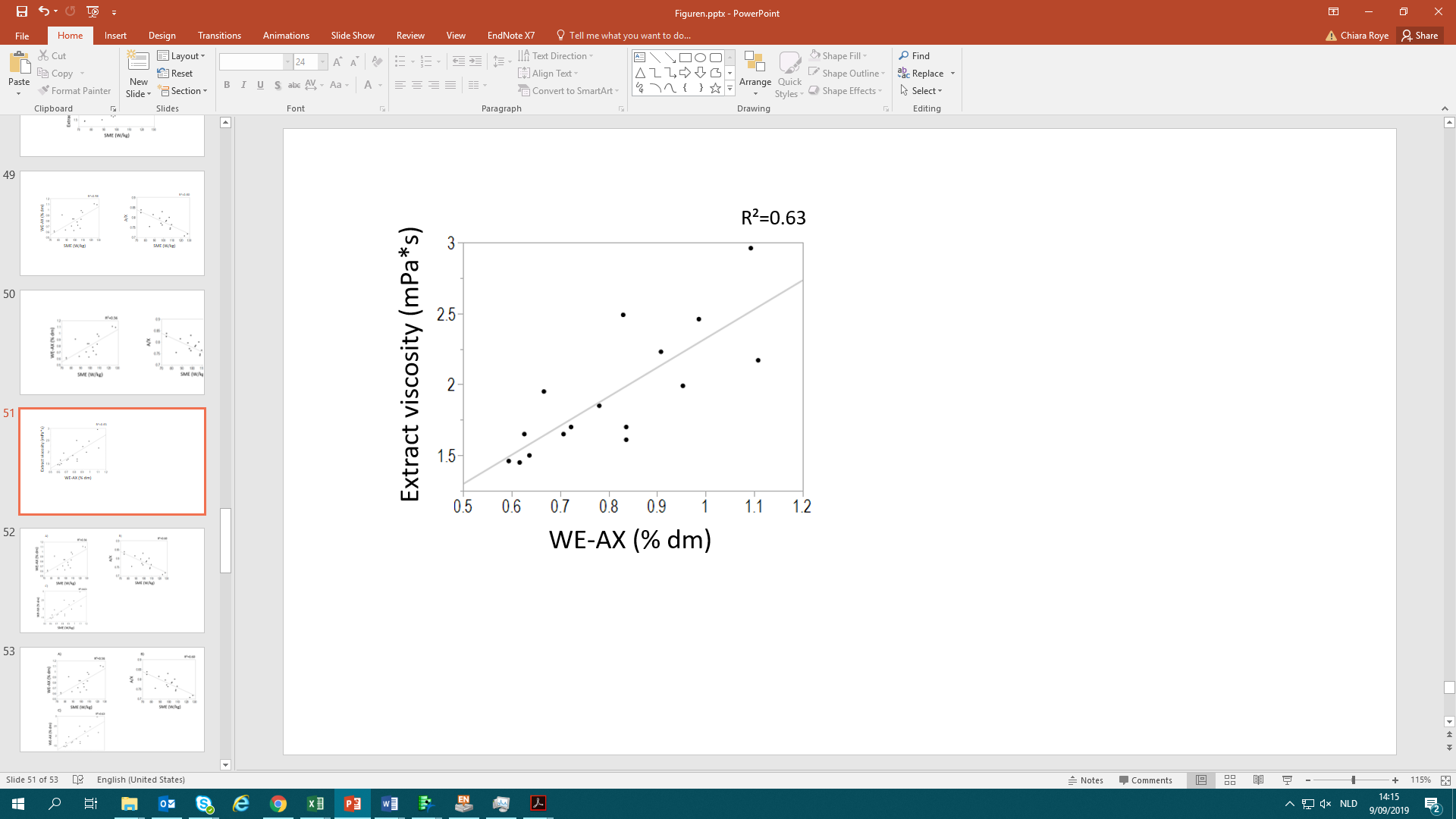


**Figure S3**: Plot of extract viscosity as a function of water-extractable arabinoxylan (WE-AX) content. The plot has an R² of 0.63.
